# Quantitative Assessment of Visible Nigrosome‐1 in Patients with Parkinson's Disease

**DOI:** 10.1002/mdc3.70547

**Published:** 2026-02-03

**Authors:** Maria Eugenia Caligiuri, Ilaria Chimento, Marida De Maria, Emma Biondetti, Jolanda Buonocore, Valerio Riccardo Aquila, Maria Celeste Bonacci, Emanuele Tinelli, Umberto Sabatini, Aldo Quattrone, Andrea Quattrone

**Affiliations:** ^1^ Neuroscience Research Center Magna Graecia University Catanzaro Italy; ^2^ Department of Neurosciences, Imaging, and Clinical Sciences University “G. D’Annunzio” of Chieti‐Pescara Chieti Italy; ^3^ Institute for Advanced Biomedical Technologies University “G. D’Annunzio” of Chieti‐Pescara Chieti Italy; ^4^ Institute of Neurology, Department of Medical and Surgical Sciences Magna Graecia University Catanzaro Italy; ^5^ Institute of Neuroradiology, Department of Medical and Surgical Sciences Magna Graecia University Catanzaro Italy

**Keywords:** area, Nigrosome, Parkinson's disease, QSM, SWI, volume

## Abstract

**Background:**

The nigrosome‐1 (N1) sign on susceptibility‐weighted imaging (SWI) typically disappears in Parkinson's disease (PD), though some patients can show uni−/bilaterally preserved N1.

**Objective:**

Investigating whether visible nigrosomes in PD patients differ from those of healthy subjects (HC).

**Methods:**

Forty‐eight PD and 35 HC underwent 3 T‐MR‐SWI. The N1 was visually assessed, and visible N1 were segmented to calculate volume, area and susceptibility values. These metrics were investigated for distinguishing between visible nigrosomes‐1 of PD patients and HC.

**Results:**

Among PD patients, 16 had bilateral N1 loss and 32 had unilaterally (*n* = 27) or bilaterally (*n* = 5) preserved N1. The visible N1 were significantly smaller in PD than in HC (*P* < 0.001), while having similar susceptibility values (*P* = 0.251). N1 area and volume showed high performance (AUC: 0.97–0.98) in distinguishing PD from HC.

**Conclusion:**

The measurement of volume and area of N1 region may complement visual assessment on SWI to optimize diagnostic performance in PD.

## Introduction

Parkinson's disease (PD) is caused by the loss of dopaminergic neurons in the substantia nigra (SN) pars compacta (SNc).^1^, ^2^, ^3^ These cells contain neuromelanin (NM), a dark pigment believed to have a neuro‐protective function against the toxicity of iron‐mediated oxidative processes.^4^ In the SNc, it is possible to identify five clusters of high NM concentration, called nigrosomes.^5^ The nigrosome‐1 (N1), located in the dorsolateral region of the SNc, is the most affected one in PD.^5^, ^6^ N1 appears as a hyperintense region in the dorsolateral SN surrounded by a bifurcated hypointense tail, visible on axial susceptibility‐weighted imaging (SWI) MR images, termed “nigrosome‐1 sign”.^7^, ^8^, ^9^, ^10^ This sign is clearly visible in healthy individuals (HC), while it often disappears in PD patients, reflecting the loss of dopaminergic neurons, NM depigmentation and increased iron deposition.^7^, ^8^, ^9^, ^10^, ^11^, ^12^ Several studies evaluated the potential of the N1 sign as a biomarker for distinguishing PD patients from HC.^7^, ^8^, ^9^, ^10^, ^11^ Most authors focused on N1 qualitative visual assessments (ie, visible bilaterally, visible unilaterally, not visible), and a unilateral or bilateral N1 loss is considered suggestive of PD.^7^, ^8^, ^9^, ^10^, ^11^ Recent advancements in artificial intelligence technology led to deep learning methods to automatically rate the N1 sign as abnormal or normal, performing similarly to expert neuroradiologists, of potential future relevance.^13^, ^14^ However, it is unclear if nigrosomes which are still visible (at least unilaterally) in PD patients already display signs of iron accumulation or are indistinguishable from those of HC. Indeed, very few data exist on quantitative assessment of N1 imaging features,^14^, ^15^ since previous studies focused on the whole substantia nigra or SNc without segmenting and analyzing visible nigrosomes.^11^, ^16^, ^17^, ^18^, ^19^, ^20^, ^21^

In this study, we performed a qualitative and quantitative assessment of the structural and susceptibility properties of N1 in PD patients using SWI and Quantitative susceptibility mapping (QSM). We focused on PD patients who had visible N1 at least unilaterally, to test whether visible nigrosomes‐1 in PD differ from those of HC and investigate the usefulness of N1 quantitative features for distinguishing PD patients from HC in the absence of the typical qualitative N1 sign loss.

## Materials and Methods

Fifty‐three PD patients and 38 HC were consecutively enrolled at our institution. The clinical diagnosis of PD was performed according to international diagnostic criteria.^2^ All patients underwent a neurological examination off‐medication overnight including the Movement‐Disorders‐Society‐revised Unified Parkinson's Disease Rating Scale‐pars‐III (MDS‐UPDRS‐III)^22^ and the Hoehn‐Yahr stage (HY). All participants underwent brain 3T‐MRI, and a PD subgroup underwent dopamine imaging (DaTscan). Subjects without any neurological disease, independent in daily life activities, were enrolled as HC. Further information is provided in supplementary material.

### MRI Acquisition and Processing

Brain 3T‐MRI included volumetric T1‐weighted, T2‐FLAIR, multi‐echo SWI for QSM analysis, and a second SWI acquisition to optimize N1 visualization (supplementary material). The visual assessment of N1 of each side was performed independently by two expert raters blinded to clinical diagnosis, and the inter‐rater agreement was calculated. A third rater was involved to reach a consensus in case of discrepant evaluations. Subsequently, visible N1 were manually segmented on optimized SWI images on three consecutive caudo‐cranial slices just below the red nucleus. The whole segmentation was used for volume and QSM calculation; moreover, for each N1, the slice with the best exposure was identified to measure N1 area (Fig. S1). After 2 weeks, all SWI images and segmentations were re‐checked by the same raters to ensure the accuracy of evaluation and segmentation procedures. Volumes (mm^3^), areas (mm^2^) and regional χ values (parts per billion, ppb) of the segmented N1 were calculated using FSL's fslstats. For QSM analysis, phase unwrapping was performed on multi‐echo SWI using ROMEO^23^; subsequently, a brain mask was calculated from the 5th‐TE magnitude image using FSL BET^24^ and background fields were removed from the B0 map calculated by ROMEO using VSHARP^25^; finally, local field‐to‐magnetic susceptibility inversion was performed using Tikhonov regularization and L‐curve optimization.^26^ Three PD patients and two HC were excluded due to severe motion artifacts on SWI.

### Statistics

Statistical analyses are described in Supplementary Material.

## Results

The final cohort included 50 PD and 36 HC (Fig. S2). For each subject, N1 visibility on each side was assessed by two experts, and the inter‐rater agreement for N1 sign presence/absence was 89.7% in the PD cohort and 93.4% in the whole population. In most (48/50, 96.0%) PD patients and HC (35/36, 97.2%), both N1 were rated as visible/absent, while two patients and one HC were deemed not evaluable and excluded from the analyses. PD patients were then stratified into those with uni−/bilaterally visible N1 (*n* = 32) and those with bilateral N1 loss (*n* = 16) (Table 1, Fig. S2). The qualitative unilateral/bilateral N1 loss showed 89.6% sensitivity (43/48), 94.3% specificity (33/35) and 91.6% accuracy (76/83) in distinguishing PD from HC. Among PD subgroups based on N1 visibility, no differences were observed in age or disease duration/severity (Table 1).

**TABLE 1:** Demographic and clinical data of patients with Parkinson's disease, stratified according to the presence or absence of nigrosome‐1 sign on susceptibility weighted MR imaging

Data	HC (*N* = 35)	PD (*N* = 48)	PD with bilateral loss of N1 (N = 16)	PD with uni−/bilaterally visible N1 (*N* = 32)	*P*‐value
Sex, (M/F)	14/21	31/17	8/8	23/9	0.201^a^ ^,^ *
Age at examination, ys^b^	49.4 ± 15.3	66.3 ± 9.6	66.2 ± 11.2	66.3 ± 8.9	0.710^c^ ^,^ ^#^
Disease duration, ys^b^	‐	5.5 ± 4.6	5.6 ± 4.2	5.5 ± 4.9	0.725^d^
MDS‐UPDRS score^b^	‐	42.1 ± 22.3	45.9 ± 25.5	40.2 ± 20.7	0.554^d^
MDS‐UPDRS‐III score^b^	‐	22.1 ± 13.2	24.6 ± 12.7	20.8 ± 13.5	0.228^d^
H‐Y score^e^	‐	1.25 (1–4)	1.5 (1–4)	1.0 (1–4)	0.533^d^
DaTscan (bilat/uni/normal)	‐	26/3/0	8/0/0	18/3/0	0.999^a^

Abbreviations: PD = Parkinson's disease; N1 = nigrosome‐1; MDS‐UPDRS = Movement Disorder Society—Unified Parkinson's Disease Rating Scale; H‐Y = Hoehn and Yahr. Bilat = bilateral striatal dopaminergic deficit; uni = unilateral striatal dopaminergic deficit. *P* values refer to the comparison across the two PD patient subgroups. DaTscan was available in 29/48 PD patients.

^a^ Fisher's exact test.

^b^ Data are expressed as mean ± standard deviation.

^c^ ANOVA or Kruskal‐Wallis rank sum test, followed by post‐hoc test with Bonferroni correction.

^d^ *t*‐test or Wilcoxon rank sum test.

^e^ Data are expressed as median (range).

* PD vs HC, Fisher's test, *P* = 0.030.

^#^ PD vs HC, t‐test, *P* < 0.001.

### Quantitative Nigrosome‐1 Assessment

**Figure 1:**
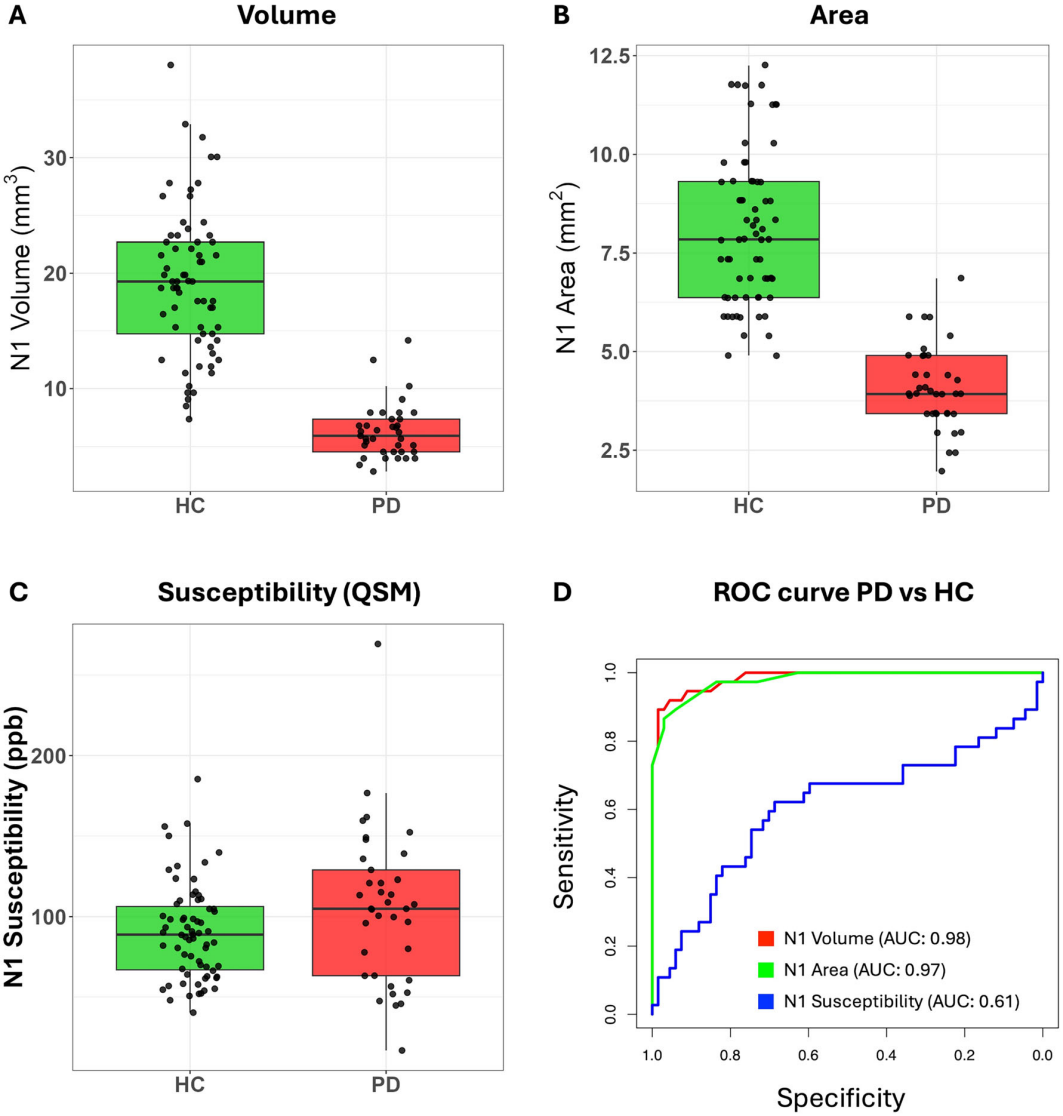
Boxplots of Nigrosome‐1 (N1) volume (A), area (B), and susceptibility (C) in patients with Parkinson's disease (PD) and healthy controls (HC). All visible N1 were considered in the analyses, including 37 nigrosomes from 32 PD patients and 67 nigrosomes from 34 HC. Volume, *P* < 0.001; Area, *P* < 0.001; Susceptibility, *P* = 0.251 in ANCOVA with age and sex as covariates. D) Receiver Operating Characteristic (ROC) curves of visible nigrosome‐1 volume (red), area (green) or susceptibility values (blue) in distinguishing visible N1 of PD from those of HC.

All visible N1 were segmented (Fig. S1) to calculate volume, area and susceptibility values. In PD patients, when the N1 was visible, its volume and area were significantly lower (*P* < 0.001, FDR‐corrected) than in HC, after correcting for age and sex (Fig. 1). Conversely, no differences in susceptibility values were observed in visible N1 between PD patients and HC (*P* = 0.251). No differences in these quantitative N1 features were found between PD patients with either one or two visible N1 on SWI. Additional analyses adjusting for total intracranial volume, using an age‐matched subset, as well as effect sizes, showed consistent results (supplementary material, Table S1).

### Classification Performances

Both N1 volume and area showed excellent classification performances (AUC: 0.98 [0.96–1.00] and 0.97 [0.94–0.99], respectively). Sensitivity and specificity were 93% (84–100%) and 97% (83–100%) for N1 volume, and 90% (77–100%) and 94% (79–100%) for N1 area in distinguishing PD patients from those of HC (Table S2). Conversely, N1 susceptibility values yielded suboptimal performances (AUC: 0.57 [0.42–0.71]), suggesting that structural data were more informative for discriminating PD from HC in cases with detectable N1 (Fig. 1D). The results were nearly identical after adjusting for age and sex (Volume, AUC: 0.98 [0.96–1.00]; Area, AUC: 0.97 [0.95–1.00], QSM, AUC: 0.58 [0.42–0.73]), demonstrating that the performances were not driven by age and sex differences. Consistent performance was obtained in distinguishing PD from HC, considering the average N1 values of both sides (Table S3).

## Discussion

This study provides a comprehensive characterization of Nigrosomes‐1, demonstrating that visible N1 were smaller in PD patients than in controls. The volume and area accurately differentiated visible N1 of PD patients from those of HC (AUC > 0.95) highlighting their usefulness in complementing visual assessment in clinical practice.

The gold standard technique to support clinical PD diagnosis is DaTscan^26^, ^27^; this procedure, however, is invasive, expensive and not widely available, limiting its widespread use. Thus, there's a growing interest in non‐invasive biomarkers predicting the DaTscan result and supporting PD diagnosis,^28^, ^29^, ^30^, ^31^, ^32^, ^33^, ^34^, ^35^ including neurophysiological assessment of rest tremor,^28^, ^29^, ^30^ substantia nigra imaging (SWI, neuromelanin‐sensitive MRI, transcranial sonography),^32^, ^33^, ^34^, ^35^ and fluid biomarkers based on alpha‐synuclein dosages or aggregation assays.^1^ In the imaging field, while conventional MRI is typically normal in PD, the N1 assessment through SWI shows substantia nigra involvement and is considered a reliable biomarker.^9^, ^10^ A recent meta‐analysis^10^ on visual N1 assessment demonstrated high diagnostic performance in differentiating PD from HC, though with some variability across studies. In PD patients, the N1 sign loss may occur either unilaterally or bilaterally, but very little is known about the characteristics of preserved nigrosomes‐1 on SWI. Most studies investigated the whole SN, demonstrating increased iron content in PD.^16^, ^17^, ^18^, ^19^, ^20^, ^21^ A few recent studies took a step forward, reporting iron accumulation in the dorsolateral part of SNc, where the N1 is located,^15^, ^36^, ^37^, ^38^ but merged data from preserved and not visible N1 making the advantage over the visual assessment unclear, not allowing to conclude on the N1 features when it is still visible on SWI. One study reported smaller N1 area in PD (compared to essential tremor),^39^ but patients with N1 loss (area = 0 mm^2^) were included in the analysis together with those with preserved N1; thus, data on visible N1 only are not available. In the current study, the qualitative N1 assessment showed good sensitivity (89.6%) and excellent specificity (94.3%) in distinguishing PD from HC; however, most PD patients had at least one preserved N1 on SWI. The DaTscan, on the other hand, showed bilaterally reduced putaminal uptake in most patients, suggesting that dopaminergic damage may precede N1 loss, in agreement with previous data.^12^ The novel aim of our study was to characterize the N1 in PD patients when it was still visible (unilaterally or bilaterally) on SWI and could be segmented by a neuroradiologist. The main finding was that “preserved” N1 of PD patients were significantly smaller than those of HC, and N1 volume or area measurements showed excellent classification performance (AUC > 0.95), representing promising quantitative biomarkers. In this context, the N1 area may be more suitable than volume for clinical practice, since it can be directly segmented on 3T‐SWI without requiring high expertise or technology. Conversely, susceptibility values showed large overlap between groups, not allowing to distinguish PD from HC nigrosomes‐1. A possible explanation was that iron may progressively accumulate from the peripheral regions toward the center of N1 in PD,^12^ leading to a progressive reduction of the size of the bright region visible on SWI (still with little iron accumulation), rather than presenting with a N1 region of preserved size but darker appearance. Longitudinal imaging studies from completely visible N1 to definite loss may shed further light on this point.

The main strength of this study was a comprehensive assessment of visible N1, including quantitative morphometric measures (area/volume) and susceptibility data. Among limitations, we acknowledge the lack of post‐mortem diagnostic confirmation, the relatively small sample size and the absence of patients with atypical parkinsonism or in the prodromal phase. While the visual N1 assessment does not distinguish PD from atypical parkinsonism, it is possible that quantitative N1 features may differ across these diseases, and future studies are warranted to clarify this point.

Overall, the quantitative assessment of nigrosome‐1 could provide insights on changes preceding the complete N1 loss on SWI images, and the N1 area may represent a simple and promising imaging biomarker for PD, complementing the visual evaluation to optimize diagnostic accuracy.

## Author Roles

(1) Research project: A. Conception, B. Organization, C. Execution; (2) Statistical Analysis: A. Design, B. Execution, C. Review and Critique; (3) Manuscript Preparation: A. Writing of the first draft, B. Review and Critique.

M.E.C.: 1A, 1B, 1C, 2A, 3A.

I.C.: 1C, 3B.

M.D.M.: 1B, 2B.

E.B.: 2C, 3B.

J.B.: 1B, 1C.

V.R.A.: 1B, 2B.

M.C.B.: 1B, 2B.

E.T.: 1B,1C, 3B.

U.S.: 1B, 1C, 3B.

Al.Q.: 1A, 2C, 3C.

An.Q.: 1A, 2C, 3C.

## Disclosure

**Ethical Compliance Statement:** All study procedures and ethical aspects were approved by the local Ethical Committee of Calabria Region. Written informed consent for the research was obtained from all the individuals participating in the study. We confirm that we have read the Journal's position on issues involved in ethical publication and affirm that this work is consistent with those guidelines.

**Funding sources and conflicts of interest:** An.Q. received funding from the Italian Ministry of Health, not related to the current research. E.B. has received funding from the European Union's Horizon Europe research and innovation programme under the Marie Skłodowska‐Curie grant agreement No 101066055—acronym HERMES. Al.Q. and M.E.C. received funding related to the current project by the Italian Ministry of University and Research (MUR), project MNESYS—A multiscale integrated approach to the study of the nervous system in health and disease (F63C22000640002—PE0000006). The funder played no role in study design, data collection, analysis and interpretation of data, or the writing of this manuscript. The other authors have no funding to declare. The sponsors listed here had no role in the design and conduct of the study; analysis and interpretation of the data; preparation, review, or approval of the manuscript and decision to submit the manuscript for publication. The other authors declared no conflicts of interest.

**Financial Disclosure/Conflict of Interest:** The authors have no conflict of interest to disclose. Al.Q. and M.E.C. received funding related to the current project by the Italian Ministry of University and Research (MUR), project MNESYS—A multiscale integrated approach to the study of the nervous system in health and disease (F63C22000640002—PE0000006). The funder played no role in study design, data collection, analysis and interpretation of data, or the writing of this manuscript.

## Supporting information


**Figure S1.** Representative susceptibility‐weighted imaging (SWI) images of two patients with Parkinson's disease and a healthy subject. The bottom part of the figure shows N1 segmentation on the most representative slice. (A) a 73‐year‐old lady with PD with bilateral N1 sign loss (duration: 4 years, MDS‐UPDRS‐III: 27, H‐Y: 2.5). (B) a 67‐year‐old gentleman with PD with unilateral N1 sign loss (duration: 3 years, MDS‐UPDRS‐III: 12, H‐Y: 1). (C) a 60‐year‐old lady with no neurological disorders.

**Figure S2.** A flowchart showing the study inclusion/exclusion procedures, and patient stratification based on visual assessment of nigrosome‐1. Among the 48 PD patients and 35 HC with a clearly visible or absent N1 on SWI, 16 PD patients and 1 HC showed bilateral N1 loss. A unilateral N1 was visible in 27 PD patients and 1 HC, resulting in 27 and 1 measurable N1, respectively. In contrast, 5 PD patients and 33 HC had bilaterally visible N1, resulting in 10 and 66 visible nigrosomes, respectively, for the quantitative analyses. SWI = susceptibility‐weighted imaging; PD = Parkinson's disease; HC = healthy controls, N1 = nigrosome‐1.

**Supplementary material** The supplementary materials include details on the cohort, inclusion/exclusion criteria, the brain MRI and DaTscan acquisition protocol, and describe in detail all the statistical analyses. The supplementary results include data on the group differences after adjustment for total intracranial volume.

**TABLE S1.** The table shows the standardized effect sizes (partial η^2^) from ANCOVA models aimed to compare N1 imaging features (volume, area and susceptibility values), including age and sex as covariates. The “Group” variable refers to the subject classification as Parkinson's disease or healthy control. Higher partial η^2^ values indicate a greater proportion of variance explained by the predictor.
**TABLE S2.** Classification performances of nigrosome‐1 volume, area and iron content in distinguishing nigrosomes‐1 of patients with Parkinson's disease those of healthy controls. The performances are shown as median and 95% confidence intervals on bootstrapping (*n* = 2000 iterations). The optimal cut‐off was established by Youden index. The performances were calculated based on 37 nigrosomes‐1 from Parkinson's disease patients (27 patients with unilaterally visible N1 and 5 patients with bilaterally visible N1) and 67 nigrosomes‐1 from healthy controls (1 subject with unilaterally visible N1 and 33 subjects with bilaterally visible N1).
**TABLE S3.** Classification performances of nigrosome‐1 volume, area and iron content in distinguishing patients with Parkinson's disease from healthy controls at the subject level. The performances are shown as median and 95% confidence intervals on bootstrapping (*n* = 2000 iterations). The optimal cut‐off was established by Youden index. The analysis was conducted at the subject level, with each subject contributing a single value (mean of left and right N1 when both were visible). A total of 48 patients with Parkinson's disease and 35 healthy controls with a clearly visible or absent N1 on SWI were included.

## Data Availability

The data that support the findings of this study are available from the corresponding author upon reasonable request.
