# Supplementary material for: Quantitative Assessment of Visible Nigrosome‐1 in Patients with Parkinson's Disease
Source: Mov Disord Clin Pract. 2026 Feb 3;13(8):1951–7. doi: 10.1002/mdc3.70547 (PMC13339310; doi:10.1002/mdc3.70547)
Supplement: Supplementary file 2 [file MDC3-13-1951-s003.docx]

**Supplementary Table 1.** Standardized effect sizes (partial η²) from ANCOVA models including age and sex as covariates.

| **Data** | **Parameter** | **partial η²** |
| --- | --- | --- |
| N1 Volume | Group | **0.72** |
|  | Age | 0.01 |
|  | Sex | 0.01 |
| N1 Area | Group | **0.67** |
|  | Age | 0.05 |
|  | Sex | 0.0001 |
| N1 Susceptibility | Group | 0.03 |
|  | Age | 0.12 |
|  | Sex | 0.07 |

Abbreviations: N1 = nigrosome-1.

Partial η² values were obtained from ANCOVA models including Group, Age, and Sex as predictors. Higher partial η² values indicate a greater proportion of variance explained by the predictor.

**Supplementary Table 2.** Classification performances of nigrosome-1 volume, area and iron content in distinguishing nigrosomes-1 of patients with Parkinson’s disease those of healthy controls.

| **Data** | **N1 Volume** | **N1 Area** | **N1 Susceptibility** |
| --- | --- | --- | --- |
| AUC | 0.98 (0.96 - 1) | 0.97 (0.94 – 0.99) | 0.57 (0.42 - 0.71) |
| Sensitivity | 0.93 (0.84 -1) | 0.90 (0.77 - 1) | 0.48 (0.24 - 0.69) |
| Specificity | 0.97 (0.83 - 1) | 0.94 (0.79 - 1) | 0.83 (0.66 – 0.97) |
| Accuracy | 0.95 (0.87 - 0.99) | 0.93 (0.85 - 0.97) | 0.72 (0.63 - 0.80) |

Abbreviations: N1 = nigrosome-1; AUC = Area under the ROC curve.

The performances are shown as median and 95% confidence intervals on 2000 bootstraps. The optimal cut-off was established by Youden index. The performances were calculated based on 37 nigrosomes-1 from Parkinson’s disease patients (27 patients with unilaterally visible N1 and 5 patients with bilaterally visible N1) and 67 nigrosomes-1 from healthy controls (1 subject with unilaterally visible N1 and 33 subjects with bilaterally visible N1).

**Supplementary Table 3.** Classification performances of subject-level nigrosome-1 volume, area and iron content in distinguishing patients with Parkinson’s disease from healthy controls at the subject level.

| **Data** | **N1 Volume** | **N1 Area** | **N1 Susceptibility** |
| --- | --- | --- | --- |
| AUC | 0.99 (0.98 - 1) | 0.99 (0.96 - 1) | 0.59 (0.44 - 0.73) |
| Sensitivity | 0.97 (0.91 - 1) | 0.97 (0.84 - 1) | 0.62 (0.31 - 0.81) |
| Specificity | 0.97 (0.88 - 1) | 0.97 (0.88 - 1) | 0.76 (0.59 - 0.97) |
| Accuracy | 0.97 (0.92 - 1) | 0.97 (0.91 - 1) | 0.68 (0.59 - 0.79) |

Abbreviations: N1 = nigrosome-1; AUC = Area under the ROC curve.

The performances are shown as median and 95% confidence intervals on 2000 bootstraps. The optimal cut-off was established by Youden index. The analysis was conducted at the subject level, with each subject contributing a single value (mean of left and right N1 when both were visible). A total of 48 patients with Parkinson’s disease and 35 healthy controls with a clearly visible or absent N1 on SWI were included.
