# Supplementary figures and images for: Quantitative Assessment of Visible Nigrosome‐1 in Patients with Parkinson's Disease
Source: Mov Disord Clin Pract. 2026 Feb 3:10.1002/mdc3.70547. Online ahead of print. doi: 10.1002/mdc3.70547 (PMC13339310; doi:10.1002/mdc3.70547)

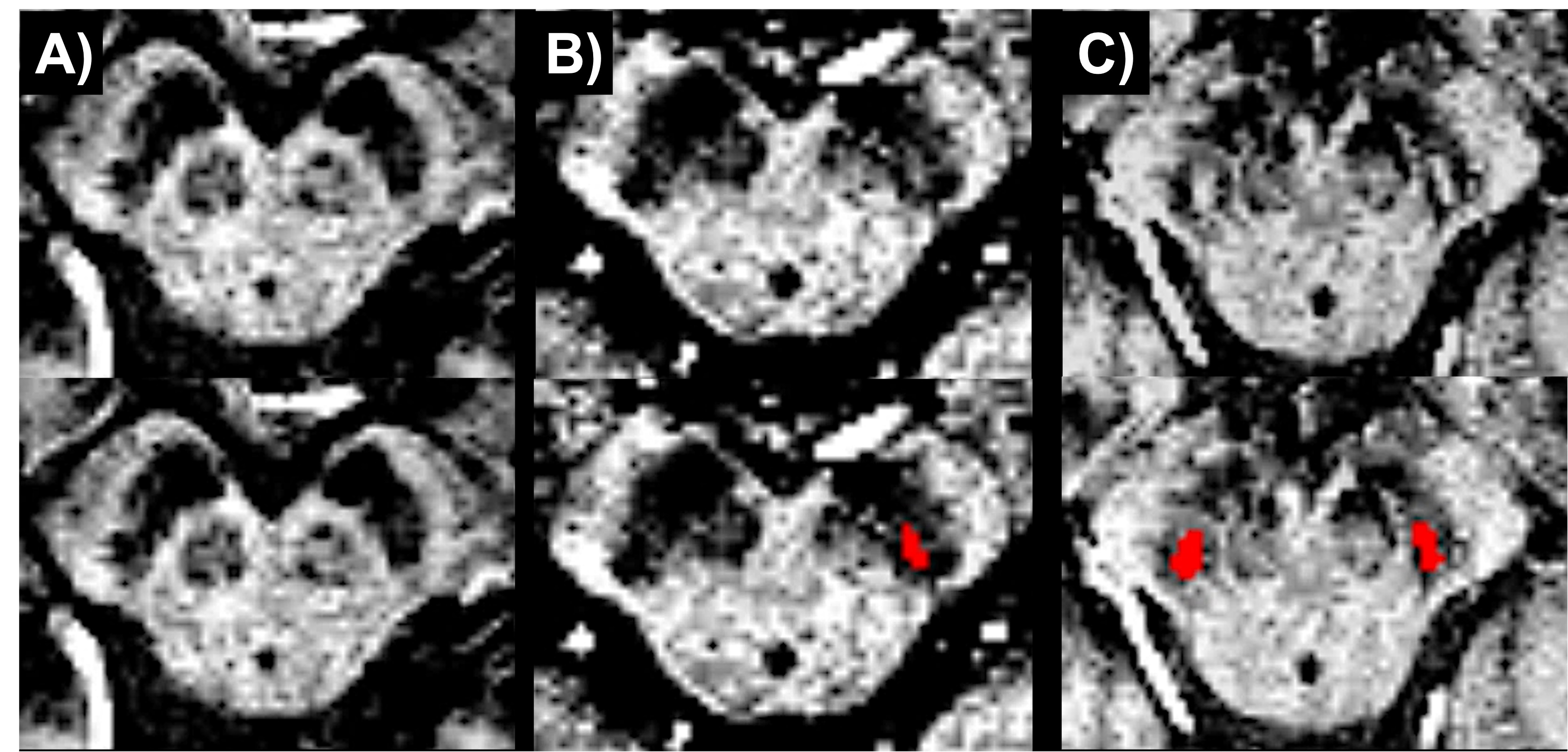

Supplement: Supplementary file 1 — Figure S1. Representative susceptibility‐weighted imaging (SWI) images of two patients with Parkinson's disease and a healthy subject. The bottom part of the figure shows N1 segmentation on the most representative slice. (A) a 73‐year‐old lady with PD with bilateral N1 sign loss (duration: 4 years, MDS‐UPDRS‐III: 27, H‐Y: 2.5). (B) a 67‐year‐old gentleman with PD with unilateral N1 sign loss (duration: 3 years, MDS‐UPDRS‐III: 12, H‐Y: 1). (C) a 60‐year‐old lady with no neurological disorders. [file MDC3-9999-0-s002.tif]

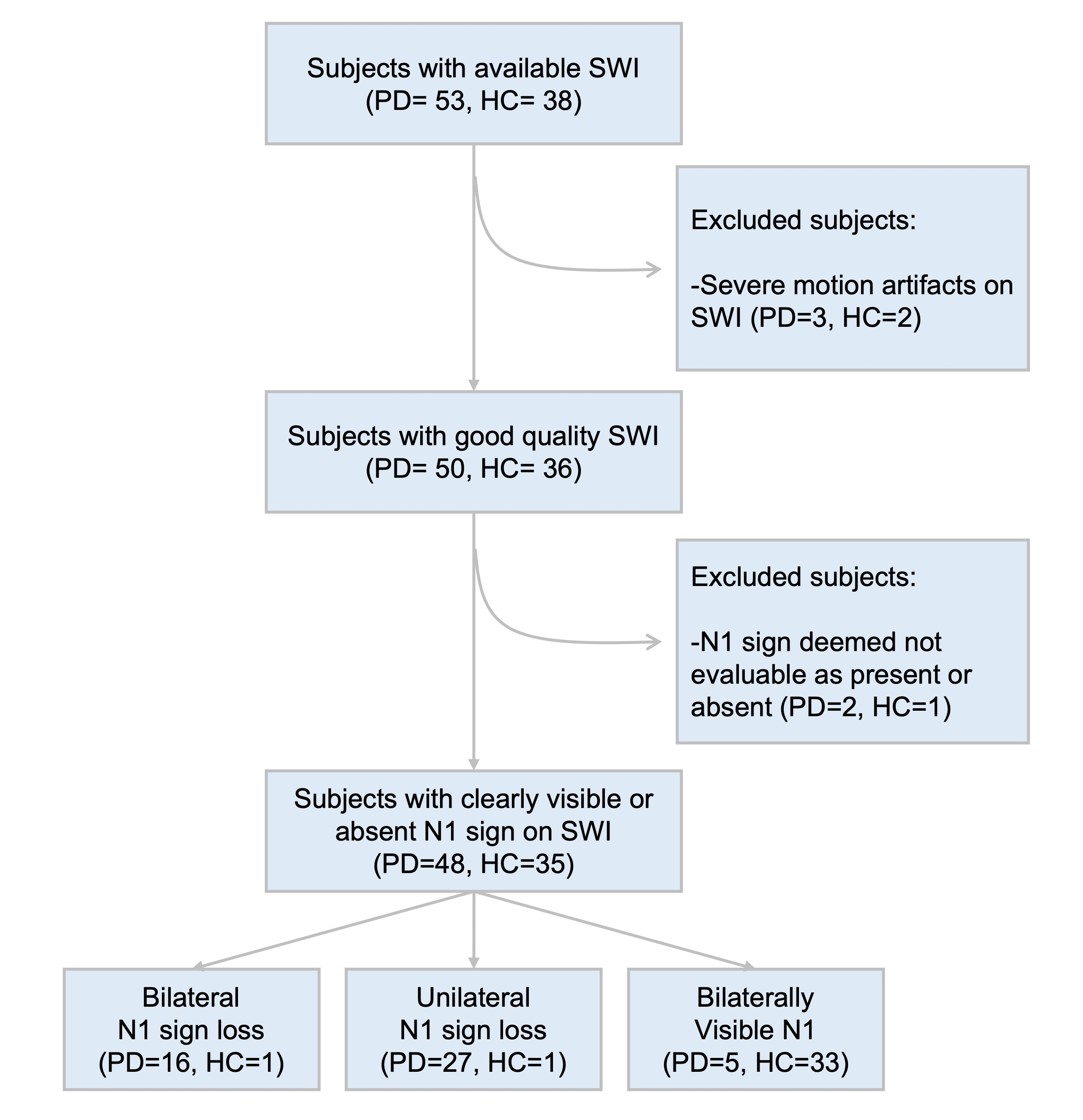

Supplement: Supplementary file 2 — Figure S2. A flowchart showing the study inclusion/exclusion procedures, and patient stratification based on visual assessment of nigrosome‐1. Among the 48 PD patients and 35 HC with a clearly visible or absent N1 on SWI, 16 PD patients and 1 HC showed bilateral N1 loss. A unilateral N1 was visible in 27 PD patients and 1 HC, resulting in 27 and 1 measurable N1, respectively. In contrast, 5 PD patients and 33 HC had bilaterally visible N1, resulting in 10 and 66 visible nigrosomes, respectively, for the quantitative analyses. SWI = susceptibility‐weighted imaging; PD = Parkinson's disease; HC = healthy controls, N1 = nigrosome‐1. [file MDC3-9999-0-s001.tif]
