# Supplementary material for: Quantitative Assessment of Visible Nigrosome‐1 in Patients with Parkinson's Disease
Source: Mov Disord Clin Pract. 2026 Feb 3:10.1002/mdc3.70547. Online ahead of print. doi: 10.1002/mdc3.70547 (PMC13339310; doi:10.1002/mdc3.70547)
Supplement: Supplementary file 3 — Supplementary material The supplementary materials include details on the cohort, inclusion/exclusion criteria, the brain MRI and DaTscan acquisition protocol, and describe in detail all the statistical analyses. The supplementary results include data on the group differences after adjustment for total intracranial volume. [file MDC3-9999-0-s003.docx]

**Supplementary Methods**

*Patients*

Fifty-three PD patients and 38 HC were consecutively enrolled at the Institute of Neurology, University Magna Graecia of Catanzaro, Italy, between January 2023 and October 2024. The clinical diagnosis of PD was performed according to most recent international diagnostic criteria.^2^ Exclusion criteria were clinical features suggestive of other diseases, and MRI abnormalities such as neoplasia, radiological signs typical of other diseases (Progressive Supranuclear Palsy, Multiple system atrophy, Normal Pressure Hydrocephalus), lacunar infarcts in the basal ganglia or diffuse subcortical vascular lesions. Subjects without any neurological disease, independent in daily life activities, were enrolled as HC. Exclusion criteria for HCs were prodromal signs of PD, as clinical suspicion of hyposmia or clinical suspicion of rapid eye movement (REM) sleep behavior disorder (RBD). All patients were also clinically followed for two years to support diagnostic stability and exclude atypical parkinsonian syndromes. All study procedures and ethical aspects were approved by the local Ethical Committee of Calabria Region. Written informed consent for the research was obtained from all the individuals participating in the study.

*MRI acquisition protocol*

All participants underwent 3T brain MRI (Biograph mMR, Siemens Healthcare, Erlangen, Germany) using a 16-channel PET-transparent head/neck coil. The protocol comprised i) Three-dimensional T1-weighted magnetization-prepared rapid acquisition gradient-echo sequence (MPRAGE, 176 sagittal planes, 256 × 247 mm^2^ field of view, voxel size 1 × 1 × 1 mm^3^, TR/TE/TI=2300/2.34/900 msec, flip angle 8°, TA = 5′12″); ii) T2-FLAIR (160 sagittal planes, 242 x 227 mm2 field of view, voxel size 0.5 x 0.5 x 1.0 mm^3^, TR/TE/TI=5000/367/1600 msec, TA = 5’27’’); iii) multi-echo susceptibility weighted imaging (SWI, 56 transverse planes centered on the midbrain, parallel to the bi-commissural line, voxel size 0.7 × 0.7 × 1.2 mm^3^, TR=50 msec, five TEs=5.88/13.62/21.62/29.62/37.96 ms, 220 x 213 mm^2^ field of view, TA = 6:23); iv) a second SWI acquisition to optimize N1 visualization using TR=29 msec, TE=18 msec and the same voxel size and field of view. For quantitative susceptibility mapping (QSM) analysis, the two SWI datasets underwent skull stripping using FSL’s BET. The qualitative SWI image, on which N1 was visually identified and segmented, was rigidly co-registered to the multi-echo SWI magnitude image used for QSM reconstruction. The resulting transformation matrix was then applied to transfer the N1 segmentations into the QSM space, ensuring accurate spatial correspondence between modalities and reliable extraction of susceptibility values from the N1 region. Images with severe motion artifacts, excessive noise, or insufficient contrast were excluded.

*Dopamine imaging assessment*

DaTscan images were acquired with an INFINIA GE Hawkeye (Milwaukee, WI, USA) without scatter and attenuation correction, and were reconstructed using OSEM algorithm (two iterations, ten subsets). Qualitative analysis was used to classify the DaTscan as normal or abnormal. The visual assessment was performed according by experienced nuclear physicians who were blinded to the patients’ diagnosis and clinical data.

*Statistical analysis*

Imaging features were compared across groups using analysis of covariance (ANCOVA) with age and sex as covariates. To evaluate the impact of inter-individual anatomical variability, additional ANCOVA models were fitted including total intracranial volume (TICV) as an extra covariate. TICV was obtained from automated segmentation of T1-weighted images, and analyses were performed on subject-level metrics (mean N1 volume, area, and QSM values across hemispheres).

To control for testing multiple imaging metrics (volume, area, QSM), p-values from ANCOVA models were corrected using the false discovery rate (FDR). Statistical significance was set at p < 0.05 after correction. Standardized effect sizes (η² and partial η²) were calculated using the effectsize R package to quantify the magnitude of group effects.

Because age differed substantially between groups, an additional sensitivity analysis was performed on an age-matched subset, restricted to the overlapping age range between PD and HC. All ANCOVA models were repeated within this subset to assess the robustness of group differences.

Associations between N1 imaging features and clinical severity measures (MDS-UPDRS total, MDS-UPDRS III, disease duration, and Hoehn–Yahr stage) were assessed using Spearman’s rank correlation.

Classification performances of N1 imaging metrics (volume, area, susceptibility) for distinguishing visible N1 in PD patients from those of HC were evaluated using the pROC package. Sensitivity, specificity, accuracy, and area under the ROC curve (AUC) were obtained with 95% confidence intervals computed through non-parametric bootstrapping (n = 2,000 iterations). To assess the influence of demographic factors, ROC analyses were also repeated using residualized imaging values after regressing out age and sex in the HC group. In addition, classification analyses were performed at the individual subject level, using the mean N1 metrics across hemispheres, which replicated the same results obtained from nigrosome-level analyses.

All statistical analyses were performed using R (version 4.0.2).

**Supplementary Results**

*Effect of TICV adjustment*

Including total intracranial volume (TICV) as a covariate in the ANCOVA models did not change the primary results. Both N1 volume and N1 area remained significantly reduced in PD compared with HC after adjusting for age, sex, and TICV (p<0.001).

TICV contributed modestly to the variance in N1 volume (p = 0.019), but its inclusion did not diminish the group effect, indicating that the observed reductions in N1 morphometric measures primarily reflect disease-related changes rather than interindividual anatomical variability.

In line with the main findings, QSM values continued to show no significant group difference after controlling for TICV (p = 0.34).
