# Supplementary material for: Quantitative Assessment of Visible Nigrosome‐1 in Patients with Parkinson's Disease
Source: Mov Disord Clin Pract. 2026 Feb 3:10.1002/mdc3.70547. Online ahead of print. doi: 10.1002/mdc3.70547 (PMC13339310; doi:10.1002/mdc3.70547)
Supplement: Supplementary file 4 — TABLE S1. The table shows the standardized effect sizes (partial η2) from ANCOVA models aimed to compare N1 imaging features (volume, area and susceptibility values), including age and sex as covariates. The “Group” variable refers to the subject classification as Parkinson's disease or healthy control. Higher partial η2 values indicate a greater proportion of variance explained by the predictor. TABLE S2. Classification performances of nigrosome‐1 volume, area and iron content in distinguishing nigrosomes‐1 of patients with Parkinson's disease those of healthy controls. The performances are shown as median and 95% confidence intervals on bootstrapping (n = 2000 iterations). The optimal cut‐off was established by Youden index. The performances were calculated based on 37 nigrosomes‐1 from Parkinson's disease patients (27 patients with unilaterally visible N1 and 5 patients with bilaterally visible N1) and 67 nigrosomes‐1 from healthy controls (1 subject with unilaterally visible N1 and 33 subjects with bilaterally visible N1). TABLE S3. Classification performances of nigrosome‐1 volume, area and iron content in distinguishing patients with Parkinson's disease from healthy controls at the subject level. The performances are shown as median and 95% confidence intervals on bootstrapping (n = 2000 iterations). The optimal cut‐off was established by Youden index. The analysis was conducted at the subject level, with each subject contributing a single value (mean of left and right N1 when both were visible). A total of 48 patients with Parkinson's disease and 35 healthy controls with a clearly visible or absent N1 on SWI were included. [file MDC3-9999-0-s004.docx]

**Supplementary Table 1.** Standardized effect sizes (partial η²) from ANCOVA models including age and sex as covariates.

| **Data** | **Parameter** | **partial η²** |
| --- | --- | --- |
| N1 Volume | Group | **0.72** |
|  | Age | 0.01 |
|  | Sex | 0.01 |
| N1 Area | Group | **0.67** |
|  | Age | 0.05 |
|  | Sex | 0.0001 |
| N1 Susceptibility | Group | 0.03 |
|  | Age | 0.12 |
|  | Sex | 0.07 |

Abbreviations: N1 = nigrosome-1.

Partial η² values were obtained from ANCOVA models including Group, Age, and Sex as predictors. Higher partial η² values indicate a greater proportion of variance explained by the predictor.

**Supplementary Table 2.** Classification performances of nigrosome-1 volume, area and iron content in distinguishing nigrosomes-1 of patients with Parkinson’s disease those of healthy controls.

| **Data** | **N1 Volume** | **N1 Area** | **N1 Susceptibility** |
| --- | --- | --- | --- |
| AUC | 0.98 (0.96 - 1) | 0.97 (0.94 – 0.99) | 0.57 (0.42 - 0.71) |
| Sensitivity | 0.93 (0.84 -1) | 0.90 (0.77 - 1) | 0.48 (0.24 - 0.69) |
| Specificity | 0.97 (0.83 - 1) | 0.94 (0.79 - 1) | 0.83 (0.66 – 0.97) |
| Accuracy | 0.95 (0.87 - 0.99) | 0.93 (0.85 - 0.97) | 0.72 (0.63 - 0.80) |

Abbreviations: N1 = nigrosome-1; AUC = Area under the ROC curve.

The performances are shown as median and 95% confidence intervals on 2000 bootstraps. The optimal cut-off was established by Youden index. The performances were calculated based on 37 nigrosomes-1 from Parkinson’s disease patients (27 patients with unilaterally visible N1 and 5 patients with bilaterally visible N1) and 67 nigrosomes-1 from healthy controls (1 subject with unilaterally visible N1 and 33 subjects with bilaterally visible N1).

**Supplementary Table 3.** Classification performances of subject-level nigrosome-1 volume, area and iron content in distinguishing patients with Parkinson’s disease from healthy controls at the subject level.

| **Data** | **N1 Volume** | **N1 Area** | **N1 Susceptibility** |
| --- | --- | --- | --- |
| AUC | 0.99 (0.98 - 1) | 0.99 (0.96 - 1) | 0.59 (0.44 - 0.73) |
| Sensitivity | 0.97 (0.91 - 1) | 0.97 (0.84 - 1) | 0.62 (0.31 - 0.81) |
| Specificity | 0.97 (0.88 - 1) | 0.97 (0.88 - 1) | 0.76 (0.59 - 0.97) |
| Accuracy | 0.97 (0.92 - 1) | 0.97 (0.91 - 1) | 0.68 (0.59 - 0.79) |

Abbreviations: N1 = nigrosome-1; AUC = Area under the ROC curve.

The performances are shown as median and 95% confidence intervals on 2000 bootstraps. The optimal cut-off was established by Youden index. The analysis was conducted at the subject level, with each subject contributing a single value (mean of left and right N1 when both were visible). A total of 48 patients with Parkinson’s disease and 35 healthy controls with a clearly visible or absent N1 on SWI were included.
